# Supplementary material for: lute: estimating the cell composition of heterogeneous tissue with varying cell sizes using gene expression
Source: BMC Genomics. 2025 May 1;26:433. doi: 10.1186/s12864-025-11508-x (PMC12045009; doi:10.1186/s12864-025-11508-x)
Supplement: Supplementary file 1 — Supplementary Material 1: Figure S1 | Pseudobulk simulation results from two DLPFC datasets with k=2 cell types. (A-B) Pseudobulk results from Huuki-Myers et al. (2023) [47]. (A) We estimated the cell composition for k=2 (neuron and glia) resolution using NNLS without (top row) and with (bottom row) scaling for differences in cell sizes, where the known cell composition is on the x-axis and the estimated cell composition is on the y-axis. The figure is faceted by cell types (neuron and glia) along the columns. (B) Boxplots of the absolute error (magnitude difference between the known and predicted cell composition) for the N=12 pseudobulk samples, for (top) glia and (bottom) neuron. (C-D) Pseudobulk results from Tran et al. (2021) [50]. (C) We estimated the cell composition using k=2 (neuron and glia) using NNLS without (top) and with (bottom) scaling for differences in cell sizes where the known cell composition is on the x-axis and the estimated cell composition is on the y-axis. The figure is faceted by cell types (neuron and glia) along the columns. (D) Boxplot of the error (difference between the known and predicted cell composition) for the N=3 pseudobulk samples, for (top) glia and (bottom) neuron. Diagonal lines indicate y = x and no error. Figure S2 | Pseudobulk simulation results from DLPFC datasets with k=3 cell types. (A-B) Pseudobulk results from Huuki-Myers et al. [47]. (A) Scatterplots of estimated the cell composition using k=3 (excitatory neuron, inhibitory neuron, and glia) using NNLS without (left) and with (right) scaling for differences in cell sizes; the known cell composition is on the x-axis and the estimated cell composition is on the y-axis. The figure is faceted by cell types (neuron and glia) along the rows. (B) Boxplots of the error (difference between the known and predicted cell composition) for the N=12 pseudobulk samples. (C-D) Pseudobulk results from Tran et al. (2021) [50]. (C) Scatterplots of the cell composition using k=3 (excita [file 12864_2025_11508_MOESM1_ESM.docx]

# Supplemental figures

**
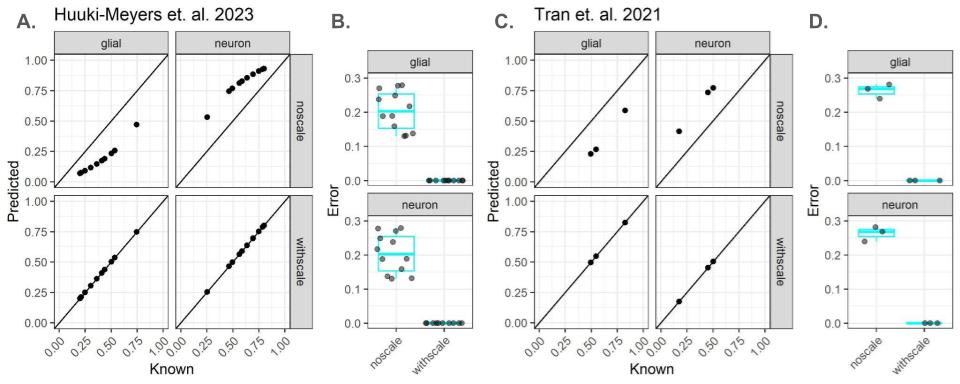
**

[**Figure S1**](#sfigu_bench_k2) | **Pseudobulk simulation results from two DLPFC datasets with *k=2* cell types**. (**A-B**) Pseudobulk results from Huuki-Myers et al. (2023) [(47)](https://sciwheel.com/work/citation?ids=14402183&pre=&suf=&sa=0). (**A**) We estimated the cell composition for *k*=2 (neuron and glia) resolution using *NNLS* without (top row) and with (bottom row) scaling for differences in cell sizes, where the known cell composition is on the *x*-axis and the estimated cell composition is on the *y*-axis. The figure is faceted by cell types (neuron and glia) along the columns. (**B**) Boxplots of the absolute error (magnitude difference between the known and predicted cell composition) for the *N*=12 pseudobulk samples, for (top) glia and (bottom) neuron. (**C-D**) Pseudobulk results from Tran et al. (2021) [(50)](https://sciwheel.com/work/citation?ids=11759316&pre=&suf=&sa=0). (**C**) We estimated the cell composition using *k*=2 (neuron and glia) using *NNLS* without (top) and with (bottom) scaling for differences in cell sizes where the known cell composition is on the *x*-axis and the estimated cell composition is on the *y*-axis. The figure is faceted by cell types (neuron and glia) along the columns. (**D**) Boxplot of the error (difference between the known and predicted cell composition) for the *N*=3 pseudobulk samples, for (top) glia and (bottom) neuron. Diagonal lines indicate y = x and no error.


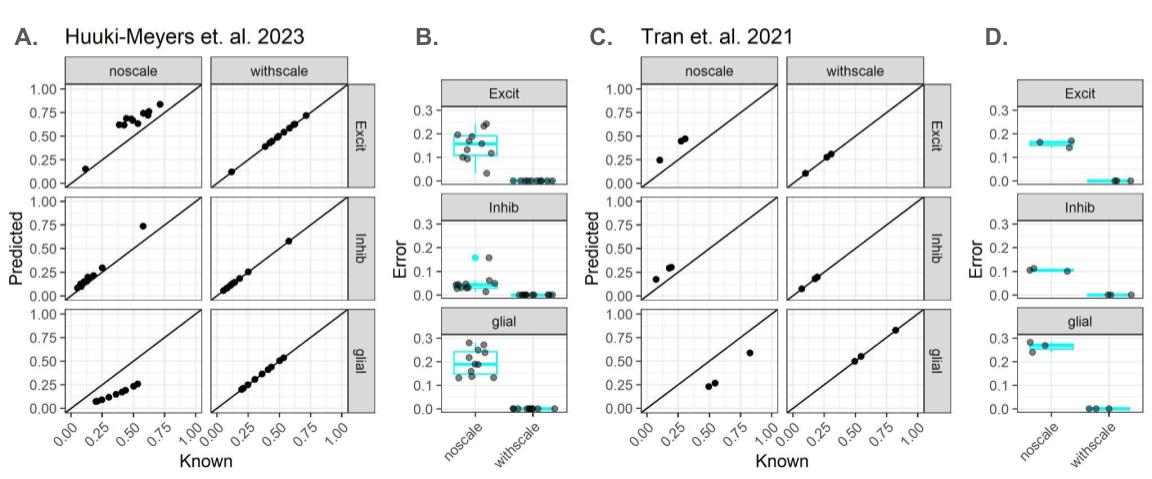


[**Figure S2**](#sfigu_bench_k3) | **Pseudobulk simulation results from DLPFC datasets with *k=3* cell types.** (**A-B**) Pseudobulk results from Huuki-Myers et al. [(47)](https://sciwheel.com/work/citation?ids=14402183&pre=&suf=&sa=0). (**A**) Scatterplots of estimated the cell composition using *k*=3 (excitatory neuron, inhibitory neuron, and glia) using *NNLS* without (left) and with (right) scaling for differences in cell sizes; the known cell composition is on the *x*-axis and the estimated cell composition is on the *y*-axis. The figure is faceted by cell types (neuron and glia) along the rows. (**B**) Boxplots of the error (difference between the known and predicted cell composition) for the *N*=12 pseudobulk samples. (**C-D**) Pseudobulk results from Tran et al. (2021) [(50)](https://sciwheel.com/work/citation?ids=11759316&pre=&suf=&sa=0). (**C**) Scatterplots of the cell composition using *k*=3 (excitatory neuron, inhibitory neuron, and glia) using *NNLS* without (left) and with (right) scaling for differences in cell sizes where the known cell composition is on the *x*-axis and the estimated cell composition is on the *y*-axis. The figure is faceted by cell types (neuron and glia) along the rows. (**D**) Boxplots of the error (difference between the known and predicted cell composition) for the *N*=12 pseudobulk samples. Diagonal lines indicate y = x and no error.


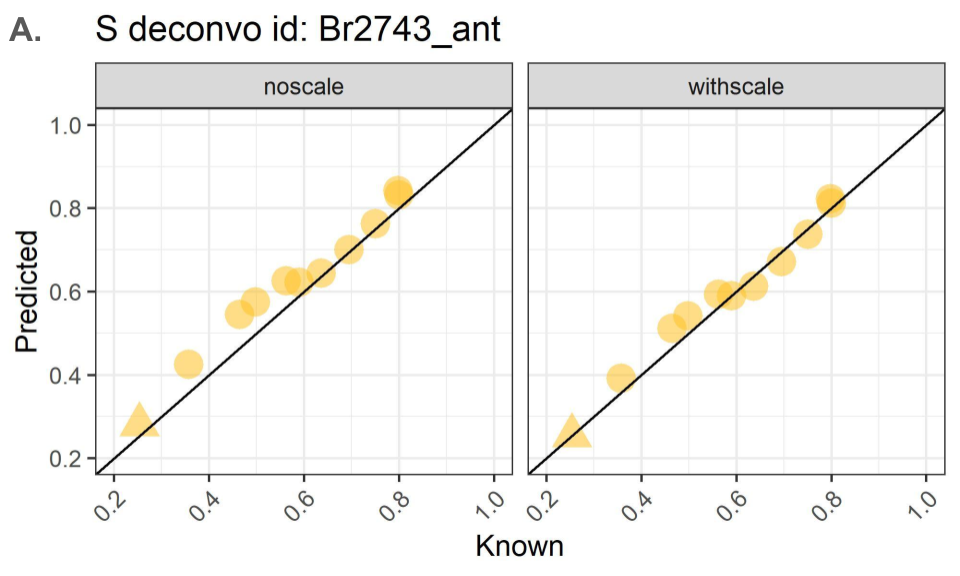

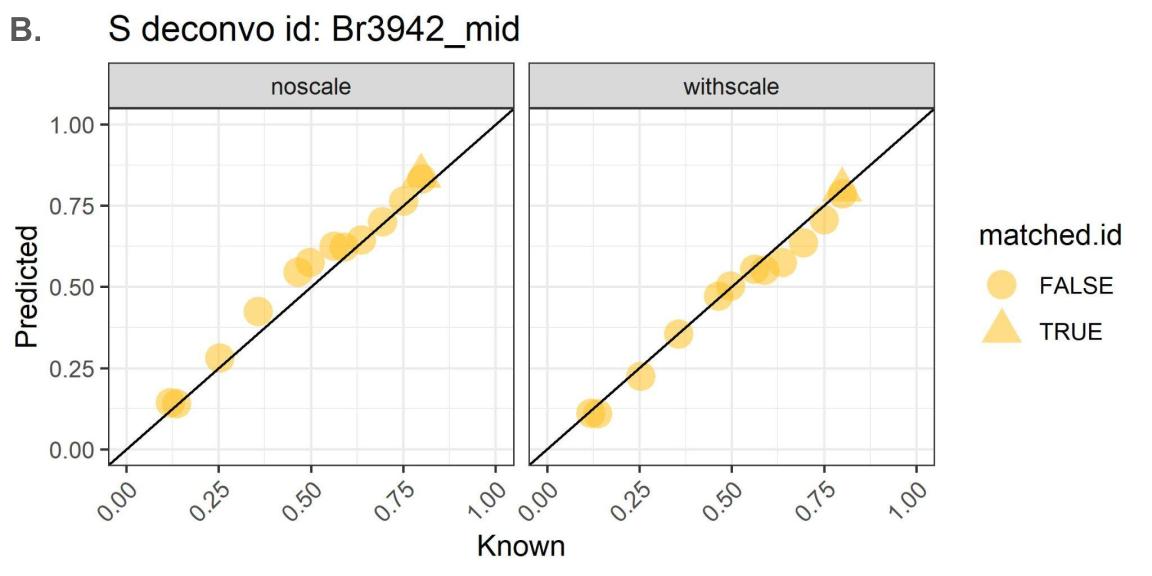


[**Figure S3**](#sfigu_rnascope_sims) **| Impact of randomly shuffling RNAScope cell scale factors in pseudobulk simulations.**

(**A**) Scatterplots of either (left panel) with or (right panel) without adjusting by cell sizes from sample with low neuron proportions (plot title, Br2743_ant). (**B**) Scatterplots of either (left panel) with or (right panel) without adjusting by cell sizes from sample with high neuron proportions (plot title, Br3942_mid, [**Table S7**](#sta_bench_donor_shuffle)). Points correspond to if the cell sizes were matched (triangle) or unmatched (circle), where references and cell scale factor arrays were calculated from DLPFC dataset [(48)](https://sciwheel.com/work/citation?ids=15527580&pre=&suf=&sa=0). Diagonal lines indicate y = x and no error.


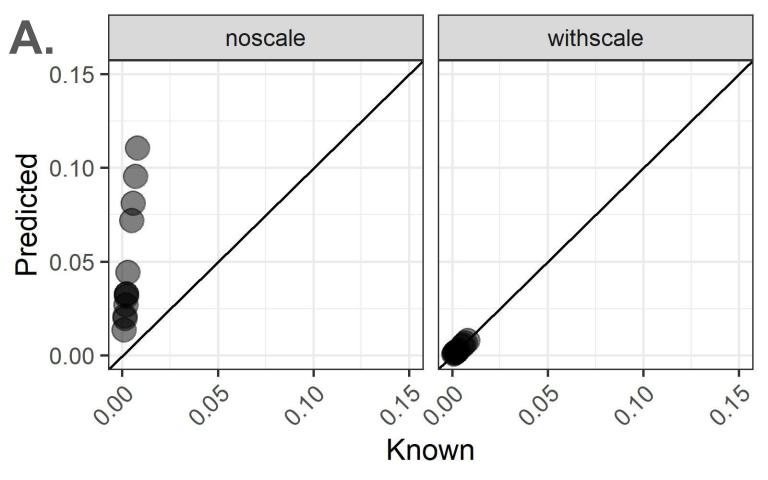

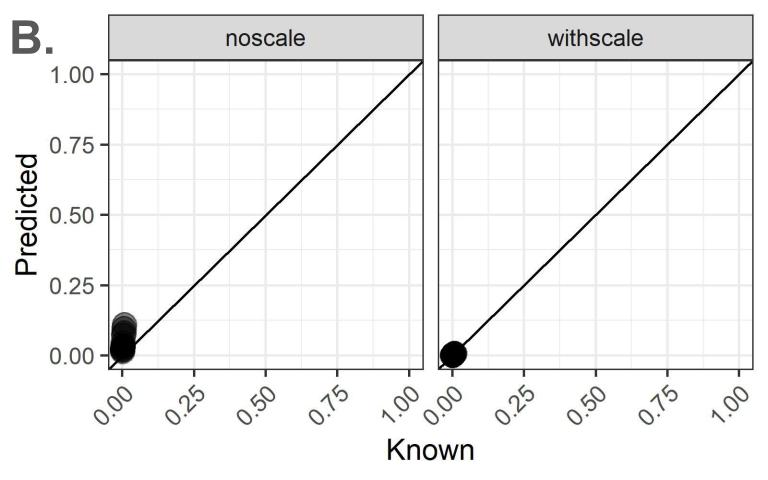


[**Figure S4**](#sfigu_pbmc) **| Deconvolution results before and after rescaling in an independent PBMC experiment.** Results are shown for *N*=12 samples from [(15)](https://sciwheel.com/work/citation?ids=6369672&pre=&suf=&sa=0) with known proportions from flow cytometry (**Methods**) at two zoom levels, either (**A**) axis maximum = 0.15 or (**B**) axis maximum = 1. Scatterplots show the (x-axis) known flow cytometry proportions versus the (y-axis) predicted proportions of Plasmablasts either (right panel) before or (left panel) after rescaling on cell size scale factors. Diagonal lines indicate y = x and no error.


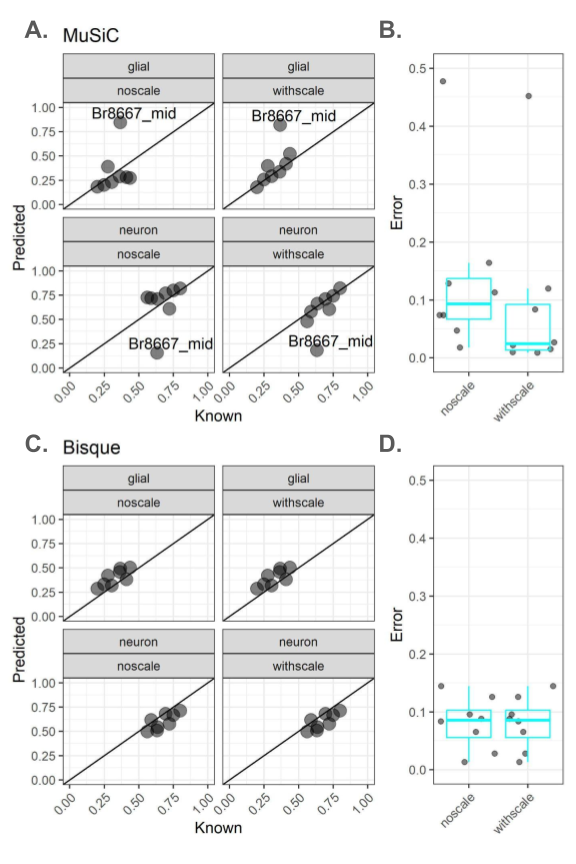


[**Figure S5**](#sfigu_real_music) **| Results of neuron predictions across deconvolution algorithms in experimental DLPFC RNA-seq samples from** [**(47)**](https://sciwheel.com/work/citation?ids=14402183&pre=&suf=&sa=0)**.** (**A**) Scatterplots show results from *MuSiC* in (points) experimental DLPFC bulk RNA-seq samples (top row) glial and (bottom row) neurons either (left column, “noscale”) without scaling or (right column, “withscale”) with scaling, with text label indicating outlying sample. Diagonal lines indicate y = x and no error. (**B**) Jittered points and quantile boxplots of (y-axis) errors by (x-axis) scaling. (**C**) Scatterplots show results from *Bisque* in (points) real bulk RNA-seq samples (top row) glial and (bottom row) neurons either (left column, “noscale”) without scaling or (right column, “withscale”) with scaling. Diagonal lines indicate y = x and no error. (**D**) Jittered points and quantile boxplots of (y-axis) errors by (x-axis) scaling.


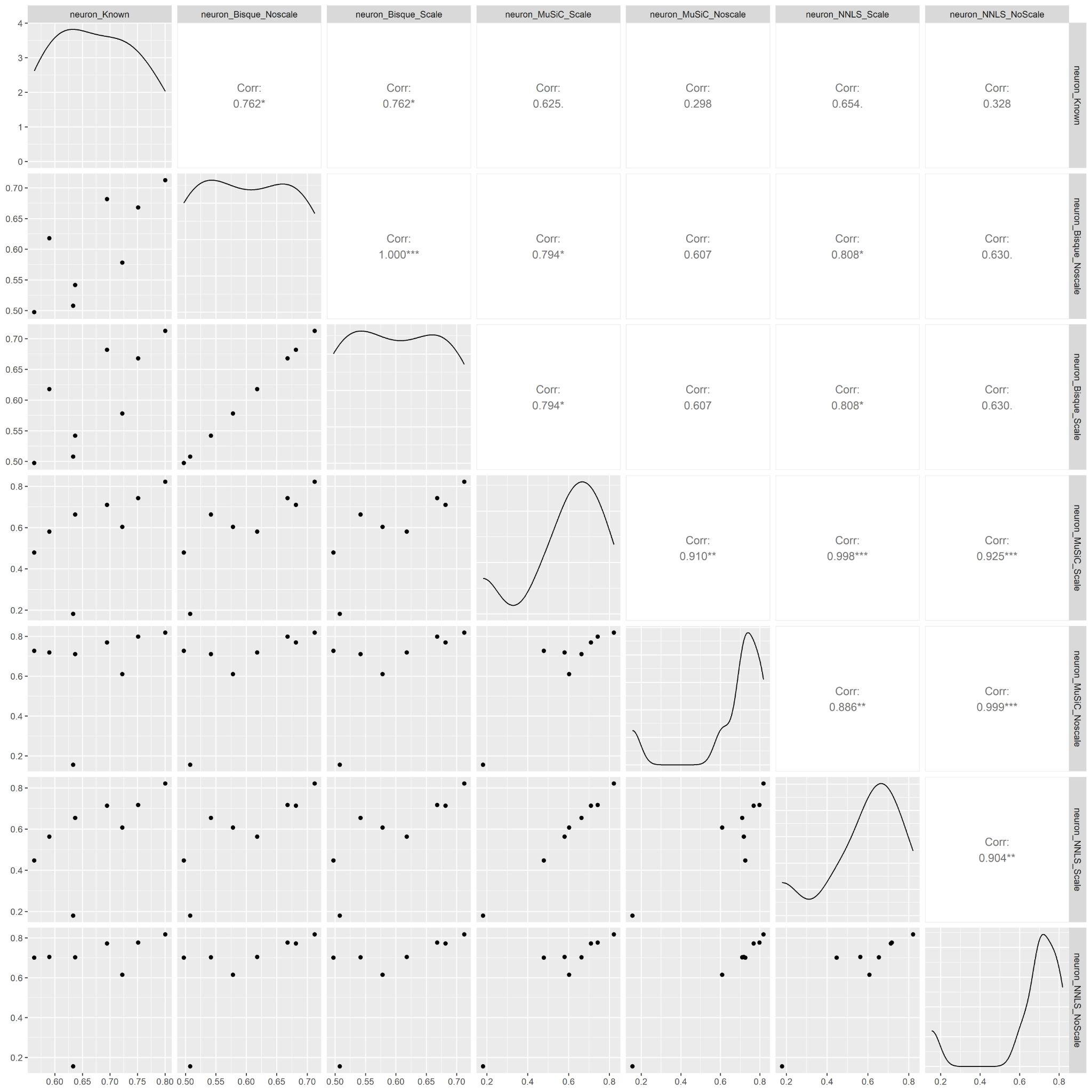


[**Figure S6**](#sfigu_correlations) **| Correlations of predicted and known neurons in experimental DLPFC bulk RNA-seq, from** [**(48)**](https://sciwheel.com/work/citation?ids=15527580&pre=&suf=&sa=0)**, for *NNLS*, *MuSiC*, and *Bisque*.** Pairs plots generated using *GGally* of known and predicted neuron proportions from multiple algorithms in real bulk RNA-seq samples from multiple preparation conditions, for neuron. Row and column labels indicate the cell type, algorithm (either “nnls” for *NNLS* [(31)](https://sciwheel.com/work/citation?ids=15497684&pre=&suf=&sa=0), “music” for *MuSiC* [(57)](https://sciwheel.com/work/citation?ids=15993176&pre=&suf=&sa=0), or “bisque” for *Bisque* [(9)](https://sciwheel.com/work/citation?ids=8798196&pre=&suf=&sa=0), or known), and condition (either scale or noscale). Text panels contain the Pearson R correlation magnitude, with asterisks indicating significance (none : 0.10 <= p; . : 0.05 < p < 0.10; * : 0.01 < p < 0.05; ** : 1.0*10^-3^ < p < 0.01; *** : p < 1.0*10^-3^).


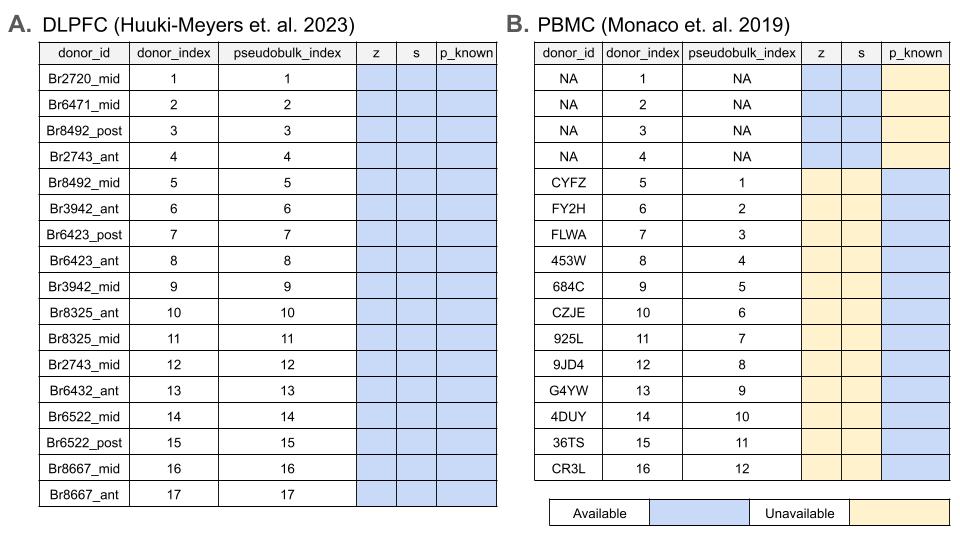


[**Figure S7**](#sfigu_pseudobulk_avail) **| Availability of samples by cohort for pseudobulk experiments in two cohorts and two tissues.** (**A**) Availability of samples for pseudobulk experiments in Huuki-Myers et al. [(47)](https://sciwheel.com/work/citation?ids=14402183&pre=&suf=&sa=0), with columns indicating (left to right) donor identifier, donor index, pseudobulk index, $Z$, $S$, and $P\_known$. (**B**) Availability of samples for pseudobulk experiments in Monaco et. al. 2019 [(51)](https://sciwheel.com/work/citation?ids=15923277&pre=&suf=&sa=0), with columns indicating (left to right) donor identifier, donor index, pseudobulk index, $Z$, $S$, and $P\_known$. Details about pseudobulk data types provided in **Methods**. Cell colors for $Z$, $S$, and $P\_known$ indicate data was either (blue) available or (yellow) unavailable for analysis
